# Water Vapor Adsorption on Biomass Based Carbons under Post-Combustion CO_2_ Capture Conditions: Effect of Post-Treatment

**DOI:** 10.3390/ma9050359

**Published:** 2016-05-12

**Authors:** Nausika Querejeta, Marta G. Plaza, Fernando Rubiera, Covadonga Pevida

**Affiliations:** Instituto Nacional del Carbón, Instituto Nacional del Carbón-CSIC, Apartado 73, Oviedo 33080, Spain; n.querejeta@incar.csic.es (N.Q.); m.g.plaza@incar.csic.es (M.G.P.); frubiera@incar.csic.es (F.R.)

**Keywords:** biomass based carbons, water vapor adsorption, CO_2_ capture

## Abstract

The effect of post-treatment upon the H_2_O adsorption performance of biomass-based carbons was studied under post-combustion CO_2_ capture conditions. Oxygen surface functionalities were partially replaced through heat treatment, acid washing, and wet impregnation with amines. The surface chemistry of the final carbon is strongly affected by the type of post-treatment: acid treatment introduces a greater amount of oxygen whereas it is substantially reduced after thermal treatment. The porous texture of the carbons is also influenced by post-treatment: the wider pore volume is somewhat reduced, while narrow microporosity remains unaltered only after acid treatment. Despite heat treatment leading to a reduction in the number of oxygen surface groups, water vapor adsorption was enhanced in the higher pressure range. On the other hand acid treatment and wet impregnation with amines reduce the total water vapor uptake thus being more suitable for post-combustion CO_2_ capture applications.

## 1. Introduction

Given the role of fossil fuels in primary energy consumption and the increasing global energy demand, carbon capture and storage (CCS) will become the strategy to follow in delivering energy security and attaining the necessary CO_2_ emission reduction targets. CCS involves three main routes to continue using fossil fuels in the transition to a low carbon energy system: post-combustion, pre-combustion, and oxyfuel capture. Post-combustion entails the separation of CO_2_ from the flue gases generated during combustion. Adsorption is one of the separation technologies that are being considered for CO_2_ capture applications [1].

The ideal post-combustion adsorbent needs to offer a series of characteristics: availability, CO_2_ selectivity, sufficient adsorption capacity, stability, ease of regeneration, and low cost. Adsorbents like activated carbon, alumina, metal oxides, zeolites and phosphates, metal organic frameworks (MOFs), microporous carbons as well as polymers and modified amines are employed [2]. The presence of water vapor, which is an inevitable component in flue gas, may negatively affect the capacity of these sorbents and reduces the availability of the active surface area. Thus, using as adsorbents zeolite 13X and alumina CDX Li *et al.* [3] showed that water could easily displace carbon dioxide adsorption. Also Liu *et al.* [4] used the Grand Canonical Monte Carlo method (GCMC) to predict the effects of water vapor and other trace gas impurities (O_2_ and SO_2_) on CO_2_/N_2_ separation properties of a subclass of MOFs that adopt zeolite structure types and showed that CO_2_ adsorption was affected by the presence of water.

Activated carbons (ACs) are one of the widely used adsorbents in many industries involving separation, purification, water treatment, energy storage, *etc.* because of their high surface areas and pore volumes [5]. The presence of heteroatoms such as oxygen, nitrogen, hydrogen, phosphorus, and sulfur on the carbon surface causes chemical heterogeneity. The most important heteroatom in activated carbons is oxygen which is usually bound to peripheral carbon atoms at the edges of the crystallites. Different functional groups can arise on the carbon surface due to the presence of oxygen. The most common functional groups are carboxylic, carbonyls, phenols, and lactones. It follows that the presence of heteroatoms determines the apparent acidity or basicity of the activated carbon surface. As a result, molecules that interact with the carbon in a specific way will be adsorbed more strongly and in greater amounts when functional groups are present. Moreover, functional groups may play a role in the sorption of nonpolar molecules by creating obstacles to physical adsorption and preventing the molecule from occupying the most energetically favorable position on the surface [6].

Carbon materials present high stability in moist conditions, which makes them appealing adsorbent candidates for post-combustion CO_2_ capture applications, in which the flue gas presents significant water content. In fact, H_2_O is generally the third most abundant component of flue gases after N_2_ and CO_2_. The actual content of H_2_O in the gas to be decarbonized will depend on the process where it originates (for example, the flue gas leaving a wet desulfurization unit in a coal power plant will be nearly saturated with water vapor) and its temperature (the lower the temperature the lower will be the absolute content of water vapor). Therefore the effect of water vapor on CO_2_ capture is an important issue that needs to be addressed [7].

The adsorption of water vapor in the pores of activated carbons is known to be dependent on the surface chemistry. Hydrophilic activated carbons have plenty of surface functional groups, which initiate predominant water adsorption through the hydrogen bonding between a water molecule and surface functional groups even at relative pressures below 0.1. On the other hand, water vapor adsorption on hydrophobic activated carbons with limited surface functionalities typically shows Type V isotherms, according to the IUPAC classification, with a large H2-type hysteresis loop [8].

Carbon adsorbents with a low carbon footprint can be produced at a low cost from a renewable and globally available source (biomass from agricultural residues or food industry by-products) through relatively simple heat treatment processes. These adsorbents are selective towards CO_2_, they can be easily regenerated, and unlike other physical adsorbents such as zeolites or MOFs, they are hydrophobic and show high stability in humid conditions [8]. Biomass based carbons derived from olive stones and almond shells have been reported as adsorbents with high adsorption capacity for carbon dioxide (4.8 mmol·g^−1^ at 101 kPa and 0 °C). Macadamia nut shell based carbon composites have been used for post-combustion carbon capture with 35% better efficiency than a honeycomb carbon fiber adsorbent [9,10,11].

The objective of this work is to study the adsorption of water vapor on a wide range of biomass based activated carbons so as to determine how to reduce the water uptake that corresponds to the presence of surface oxygen functional groups and to the microporosity in the carbons. Additionally, given the humidity of the flue gas to be treated in post-combustion CO_2_ capture systems, the role of this reduction on the CO_2_ uptake is also explored. For this purpose three different post-treatments were selected: heat treatment, acid treatment, and wet impregnation with amines. Water vapor adsorption isotherms were volumetrically measured. Each isotherm was fitted to semi-empirical and theoretical adsorption models that account for the role of surface chemistry and/or microporosity: Dubinin-Serpinsky (DS) [12], Dubinin-Astakhov (DA) [13] and Do-Junpirom-Do (DJD) models [14].

## 2. Materials and Methods

### 2.1. Materials

Olive stones (OS) were selected as precursors for the production of the departing biomass based carbons. Firstly, they were ground and sieved, and a particle size between 1 and 3 mm was selected for further treatment.

The development of the porous structure was attained by physical activation in a single-step activation procedure, using a double-jacket quartz reactor in a vertical furnace under a gas stream with an oxygen content of 3 vol% (balance N_2_) or under CO_2_. The single-step procedure has been shown to be competitive to the conventional two-step procedure [15,16,17].

Sample denoted as RN1 was obtained by loading approximately 70 g of raw biomass in a quartz reactor (I.D. 3.8 cm) and activation in 3% O_2_ with a flow rate of 370 cm^3^·min^−1^. Sample named as RN2 was similarly obtained by single-step CO_2_ activation. In both cases the heating rate used was 5 °C·min^−1^ and the temperatures of activation and holding times were set at 650 °C–300 min and 800 °C–360 min, respectively. The volatiles evolved during the preparation were collected and quenched in a bubbler immersed in an ice bath for mass balance purposes.

### 2.2. Methods for the Modification of Activated Carbon

Despite the hydrophobicity of carbons they do adsorb significant amounts of water that impact the adsorption of other species such as CO_2_ in post-combustion capture applications. The presence of functional groups on the carbon surface is mainly responsible for water vapor adsorption on carbons at low relative humidity. They act as nucleating sites for water to grow into clusters. Depending on the concentration of functional groups the adsorption of water at low pressures can be noticeable, and when the humidity is reasonably high the water adsorption capacity significantly increases and competes with CO_2_ adsorption. Likewise the presence of mineral matter on the carbon surface also contributes to increase water adsorption in the lower pressure range [18]. Ash content in ACs is not desirable, being considered an impurity inside the carbonaceous matrix. It may reduce and inhibit the surface area by filling or blocking some of the existing network of micropores. The mineral matter content in the ash is mainly ascribed to silica, aluminum, iron, magnesium, and calcium [19].

In order to limit water vapor adsorption, several techniques of surface hydrophobization and demineralization of microporous carbonaceous adsorbents have been explored. Most of the techniques developed to lower the affinity of water for AC act on the surface functionalities or on the mineral matter: depending on whether the latter are removed or modified, the so-called physical or chemical methods may be used, respectively. Conventional physical methods are usually based on thermal treatments in a flow of hydrogen or inert gas. In contrast, chemical methods are scarcely described in the literature and refer to different types of coatings: organosilicon (usually trimethylchlorosilane) in gas phase or fluorocarbon (C_2_F_4_ or C_2_F_6_) in liquid phase either by plasma surface treatments or through chemical reaction (or impregnation), ammonia and monomeric or polymeric amine species (e.g., diethylenetriamine), chlorination, *etc*. The treatment of ACs with different acids such as HCl, HF or HNO_3_ has also been explored [20,21].

#### 2.2.1. Heat Treatment

High temperature (>700 °C) heat treatment under inert atmosphere (nitrogen or helium) is used to selectively remove some of the surface oxygen functionalities from the carbon surface. The majority of these oxygen functionalities evolve in the 800–1000 °C temperature range. Heat treatment creates unsaturated surfaces as a result of thermal desorption of oxygen acidic functional groups. Several researchers have confirmed that the decomposition of oxygen functional groups at elevated temperatures increases basicity of the carbon surface. This is due to the fact that strongly acidic functionalities (such as carboxylic, anhydrides and lactones) decompose at lower temperatures, while the weakly acidic functionalities (such as carbonyl, phenol, and quinone) decompose at higher temperatures.

The temperature of heat treatment is commonly limited to ~1000 °C and the resulting material usually shows a low degree of activity for reacting with oxygen or chemical agents and a high degree of basicity [22].

In this way, the produced biomass based activated carbon RN1 was heat treated to enhance the hydrophobic character. The main goal of this post-treatment was to remove oxygen surface groups without much impact on the porosity of the carbon that determines its CO_2_ adsorption capability. For this purpose, 1 g of carbon RN1 was heat treated at 1000 °C under a flow of nitrogen for half an hour. Heat treatment was conducted in a vertical furnace at a heating rate of 15 °C·min^−1^. The produced sample is denoted as RN1P.

#### 2.2.2. Acid Treatment

Acid treatment involves chemical reactions between the acid and the mineral matter. The effects of the treatment may change the pore structure, surface functionality, and adsorption capacity. Acid treatment may introduce new functional groups although some other moieties may be lost during the treatment. These alterations on the surface functional groups will also impact the adsorption behavior. Different types of acids react differently with the ash and the available functional groups. Single acids, especially HCl, or a blend of acids, such as HCl and HNO_3_ are most commonly used to remove ash [19].

The reduction of the mineral matter content of carbon RN1 was pursued by washing the carbon with an acid solution of HCl 1 M (method adapted from Samaras *et al.* [23]). A single step approach was followed: 20 cm^3^ of the acid solution were mixed with 3 g of RN1 in a glass beaker. The mixture was stirred at 60 °C for 1 h. The treated carbon (RN1A) was then filtered off and subsequently rinsed with warm distilled water at 60 °C in order to eliminate the chloride ions dispersed over the sample surface. Finally, the carbon was dried at 100 °C for one hour in an oven under a light vacuum of 50 mbar.

#### 2.2.3. Impregnation with Amines

The most prominent method to enhance the CO_2_ adsorption capacity is promoting basic species on the surface of the activated carbon through chemical impregnation (e.g., alkanolamines) [24]. In this context, the term impregnation can be defined as the fine distribution of chemicals and/or metal particles in the pores of activated carbon [25].

In terms of post-combustion CO_2_ capture, taking into account that the flue gas contains significant water it is expected that carbon dioxide, water vapor and immobilized amines onto the carbon surface show similar reactions to liquid amines in a typical absorption process [24].

Amines with a short linear chain present primary and secondary amine groups and are known to adsorb CO_2_ via the carbamate mechanism under dry conditions. They tend to capture and stabilize the adsorbed CO_2_ between the amine molecules (intermolecular CO_2_ sorption). The sorption of CO_2_ on amine-functionalized solid sorbents proceeds predominantly by the carbamate mechanism; the formation of carbamic acid is also an alternative route that has been widely discussed in the literature (see Figure S1 in the Supplementary Materials) [26].

It has been widely reported that the CO_2_ uptake by solid amine-based systems increases in the presence of H_2_O because of the formation of bicarbonates (see Figure S2 in the Supplementary Materials).

The formation of a water film on the adsorbent particles is likely when H_2_O is present in the flue gas. In this work, diethylenetriamine (DETA) was immobilized on the biomass based activated carbon RN2 through a wet impregnation method adapted from Xu *et al.* [27]. This entailed dissolving the desired amount of DETA (viscous liquid) in 10 cm^3^ of methanol by mechanically stirring the mixture for about 15 min. The solution was then poured over 1 g of RN2, previously dried for one hour at 100 °C, and allowed to mix in a rotary evaporator for 120 min at room temperature. Subsequently, the resulting slurry was kept at 40 °C and 300 mbar under reflux for 30 min and an additional 30 min under the same conditions but without reflux. Methanol was eliminated by evaporation first without reflux at 40 °C for 30 min and then at 60 °C for an additional 90 min. To complete the drying step, the pressure was reduced to 50 mbar. The resulting samples were denoted as RN2D5 and RN2D10, where D5 and D10 represent the DETA loading used in the impregnation on a mass basis with respect to the mass of carbon (5 and 10 wt%).

### 2.3. Characterization of the Samples

#### 2.3.1. Chemical Analysis

The prepared samples were subjected to proximate and ultimate analyses in order to determine the chemical composition of the carbons.

Proximate analysis was carried out by means of thermogravimetry in a Setaram TGA24 thermogravimetric analyzer. The analysis consists of the following steps: at ambient temperature in a flow of air (50 cm^3^·min^−1^), approximately 10 mg of the sample is weighed directly into the TG crucible. The sample is heated from ambient temperature up to 100 °C at a rate of 5 °C·min^−1^ and is maintained at this temperature for 60 min. The weight loss at this stage is mainly due to moisture. The sample is further heated up to 815 °C at 15 °C·min^−1^ and maintained at this temperature for a further 30 min. The weight loss accounts for the carbon content as carbon is burnt off in an oxygen atmosphere. Finally, the residual weight may correspond to the ash content of the sample [28].

The carbon, hydrogen, and nitrogen contents were determined in a LECO CHNS-932 analyzer and the oxygen content in a LECO VTF-900 analyzer.

#### 2.3.2. Pore Structure Characteristics

Textural characterization of the samples was carried out by means of physical adsorption of N_2_ at −196 °C in a Micromeritics ASAP 2010 and adsorption of CO_2_ at 0 °C in a Micromeritics TriStar 3000. Helium density was measured in an Accupyc 1330 at 35 °C. The samples were outgassed at 100 °C under vacuum overnight prior to adsorption measurements.

The use of both adsorbates, N_2_ and CO_2_, provides complementary information about the porous texture of the samples: the adsorption of CO_2_ at 0 °C and up to 1 bar is restricted to pores narrower than 1 nm, whereas N_2_ adsorption at −196 °C covers wider pore sizes but presents diffusion limitations in the narrower pores. The total pore volume (V_p_) was calculated from the amount of N_2_ adsorbed at a relative pressure of 0.99, and the BET surface area from the Brunauer-Emmett-Teller equation [29]. The micropore volume (W_0_) and the micropore surface area (S_DR_) were determined from the Dubinin-Radushkevich (DR) [30] and Dubinin-Astakhov (DA) [31] equations assuming a density of the adsorbed phase of 0.808 cm^3^·g^−1^ for N_2_ and 1.023 cm^3^·g^−1^ for CO_2_, a cross sectional area of 0.162 nm^2^ for N_2_ and 0.187 nm^2^ for CO_2_ and finally an affinity coefficient of 0.34 for N_2_ and 0.36 for CO_2_. The average micropore width (L_0_) was calculated through the Stoeckli-Ballerini equation [32].

#### 2.3.3. Determination of Surface Oxygen Functional Groups

Surface chemistry is a critical factor in water vapor adsorption, especially at low relative pressures. In order to study the influence of the surface chemistry of the samples on water vapor adsorption, it is advisable to use different experimental techniques to characterize the carbon surface functionalities that can provide complementary information. In this case, Temperature Programmed Desorption (TPD) and Fourier Transform Infrared Spectroscopy (FTIR) were selected.

### 2.4. Adsorption Studies

Adsorption isotherms of CO_2_, N_2_, and H_2_O were collected. Water vapor adsorption isotherms were determined from 30 to 70 °C in a volumetric apparatus Quantachrome Hydrosorb 1000 HT where temperature was controlled by a Julabo thermostatic bath. Single component N_2_ and CO_2_ equilibrium adsorption isotherms were collected at 30 °C in a volumetric device, TriStar 3000 from Micromeritics where temperature was controlled by a Thermo Haake thermostatic bath. Before each measurement, samples were outgassed at 100 °C under vacuum overnight.

## 3. Results and Discussion

### 3.1. Characterization of the Samples

#### 3.1.1. Proximate and Ultimate Analysis

Analysis results of carbons RN1 and RN2 and their modified series are summarized in Table 1. Results show that acid treatment (RN1A) incorporates oxygen in the sample. The increase in the oxygen content of the samples impregnated with amines (RN2D5 and RN2D10) might be mainly ascribed to incomplete removal of methanol used as solvent. Thermal treatment results in a significant reduction of the oxygen content of RN1P. Heat treatment produces a reorganization of the carbonaceous structure, with a progressive mass loss, due to gaseous products evolution during the cracking process at high temperatures. The decrease in oxygen content from 7.4 to 4.2 wt% is attributed to the loss of surface oxygen groups incorporated during the air activation stage. The hydrogen content also decreases from 1.5 to 0.7 wt% while the nitrogen content remains practically unchanged.

Removal of mineral matter is attained for sample RN1A through acid treatment. The ability of the acid to remove different inorganic components is the main cause for this elimination. It has been suggested that the weight loss due to the use of hydrochloric acid may be ascribed to the removal of sulfides and carbonates [33].

Table 1 also shows that for the aminated samples the nitrogen content was successfully increased from 0.7 wt% up to 3.8 wt% through impregnation; it should be taken into account that the nitrogen content of the final sorbent depends not only on the amount of amine incorporated in the carbon but also on the nature of the amine itself, as has been reported elsewhere [24].

#### 3.1.2. Pore Structure Characteristics

**Figure 1:**
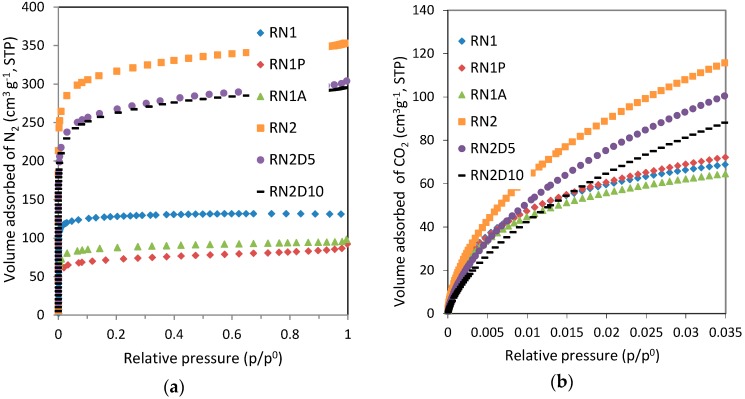
Adsorption isotherms of the prepared carbons: (**a**) N_2_ at −196 °C and (**b**) CO_2_ at 0 °C.

The N_2_ adsorption isotherms at −196 °C of the carbons are shown in Figure 1a. All the samples presented type I adsorption isotherms, characteristic of microporous materials. From the shape of the isotherm and, particularly, the open elbow in the low pressure range, it can be deduced that activation with carbon dioxide (RN2) develops both narrow and wider micropores. On the other hand, RN1 is characterized by its narrow micropores that limit the volume of N_2_ adsorbed and are responsible for the pronounced elbow at very low relative pressures.

Figure 1b represents the CO_2_ adsorption isotherms of the samples at 0 °C. The comparison of the volumes adsorbed of both adsorbates, N_2_ and CO_2_, gives an indication of the micropore ratio on each sample: CO_2_ adsorption at 0 °C evaluates the microporosity of less than 1 nm whereas micro and mesopores are assessed from N_2_ adsorption at −196 °C.

When thermal treatment is applied to RN1 to obtain RN1P, a reduction in the porous structure is observed, as a consequence of the shrinkage of the carbon matrix [34]. The heat treatment in an inert atmosphere of N_2_ at 1000 °C decreased the amount of N_2_ adsorbed by 31% but led to an increase in the adsorption of CO_2_ by 5%. The evolution of surface functional groups from RN1 during the heat treatment probably accounts for these changes in the micropore structure.

The sample subjected to acid washing, RN1A, also shows a reduction in the porosity development with respect to the parent sample RN1. This may be due to the fact that after treatment with HCl some chloride could remain chemisorbed on the surface of the sample decreasing the available micropore volume and shifting the effective sizes of the micropores to narrower widths in which N_2_ adsorption is restricted. The ability of the activated carbon to chemisorb chloride is due to the weakness of the OH-Cl hydrogen bond which ensures that there is very little impediment to complete ionic dissociation [21]. On the other hand, CO_2_ adsorption isotherms show that narrow microporosity in RN1 is only slightly affected by acid washing. The amount of CO_2_ adsorbed on RN1A is reduced by 6% with respect to the parent carbon. Acid washing of RN1 seems to slightly widen the narrow micropores. This may be a consequence of the removal of the inorganic matter in sample RN1.

The impregnation of RN2 at two loadings, 5 and 10 wt% of DETA, notably reduces the volume of available porosity for N_2_ and CO_2_ adsorption. Nevertheless, the type and sizes of the porosity seem little affected according to the shape of the isotherms. Then, it was confirmed that the amine film impregnated on the carbon surface blocks the porosity of the parent carbon to some extent.

Table 2 summarizes the textural parameters obtained from the analysis of the previously presented adsorption isotherms. Activation with carbon dioxide (RN2) leads to a better development of the texture of the starting material (1248 m^2^·g^−1^).

The modification of RN1 and RN2 results in lower BET apparent surface areas and lower total pore volume but higher average pore widths compared to the parent samples.

RN1P has the smallest S_BET_ value and pore volume of the samples, which shows that heat treatment alone acts to the detriment of textural development. However, heat treatment barely reduces the narrow micropore volume [W_0_,_CO2_].

As we have explained above, acid treatment and wet impregnation bring about a decrease in both the S_BET_ and the micropore volume [W_0_,_N2_] due to partial blockage of the pores as previously observed by other authors [21,24,35,36]. The decrease is notably more pronounced for the sample RN1A. Acid treatment and impregnation with amines also affect the narrow micropore volume [W_0_,_CO2_].

For typical activated carbons, W_0_ [CO_2_, 0 °C] is in the range of 0.2–0.3 cm^3^·g^−1^, whereas E_0_ [CO_2_, 0 °C] generally varies from 28 to 30 kJ·mol^−1^ (L_0_ ~ 0.60 nm). Although in special cases both parameters can reach slightly higher values, these guarantee an upper-bound of around 10–11 wt% CO_2_ uptake under post-combustion capture conditions as quoted in the literature [37]. As can be appreciated in Table 2. Textural parameters of the samples obtained from the N2 and CO2 adsorption isotherms the values corresponding to W_0_ and E_0_ obtained from the CO_2_ adsorption isotherms are within the ranges described above, showing a good development of the narrow microporosity. It is also interesting to note the importance of both micropore volume and micropore size distribution on the CO_2_ uptake. The average micropore width (L_0_) is a key factor of the carbon dioxide adsorption: lower values of L_0_ lead to higher adsorption potential for the carbon dioxide molecule due to the overlapping effect of the opposite walls of the micropores. Therefore activated carbons with low values of L_0_ together with a good development of the microporosity may be excellent candidates to capture CO_2_.

Likewise, water vapor adsorption is also related to the parameters W_0_ and L_0_. Despite water vapor uptake being promoted at low pressures by the content of surface oxygen functional groups, from medium to high pressures the filling of the micropores is responsible of the adsorption capacity of the activated carbon. Moreover the size of the clusters is dependent on the micropore width. Thus, one more time adsorption is governed by the development of microporosity and the micropore size distribution.

#### 3.1.3. Surface Oxygen Functional Groups

TPD tests were run on the parent and modified carbons so as to monitor the evolution of labile surface oxygen functionalities in the form of CO and CO_2_ profiles *versus* temperature (see Figure S3 in the Supplementary Materials). These curves were then integrated and deconvoluted to obtain the amount of oxygen surface groups. The results are summarized in Table 3. Decomposition of surface oxygen functionalities as CO_2_ is considerably lower than as CO. The global amount, CO + CO_2_, is indicative of the total amount of surface oxygen groups of the sample.

Heat treatment leads to higher decrease of the surface oxygen functional groups and that reduction is markedly strong in the number of surface functionalities that decompose as CO_2_. In contrast acid treatment incorporates the highest number of surface oxygen groups (both CO and CO_2_ evolving functionalities) to RN1A.

For the amine impregnated samples, RN2D5 and RN2D10, a decrease is observed in the amount of surface oxygen functionalities (CO + CO_2_) that can be explained by the film of amine on the surface of the activated carbon.

Deconvolution of the CO and CO_2_ profiles allows the estimation of the contribution of the different oxygen surface functionalities. These data are collected in Table 4 and Table 5.

Heat treatment in an inert atmosphere is not strong enough to completely eliminate carboxylic acid groups present in RN1 despite carboxylic acid groups being thermally more unstable than basic groups such as ether, carbonyl or chromene. Nevertheless, it can be observed that the amount of surface oxygen groups has been reduced and RN1P shows a more hydrophobic surface as can be inferred from the increase in the CO/CO_2_ ratio from 1.6 for RN1 up to 2.7 for RN1P.

When RN1 is acid washed an important increase in the concentrations of CO and CO_2_ evolved is observed due to the formation of new stable oxygen groups during the acid washing procedure. This also causes a strong acidification of the carbon surface as denoted by the decrease in the CO/CO_2_ ratio from 1.6 for RN1 down to 1.2 for RN1A.

Gas evolved analysis during the TPD tests of the amine impregnated carbons showed CO_2_ and CO as were to be expected for activated carbons; but NH_3_ and CH_4_ also evolved due to the decomposition of the amine coating (See Figure S4 in the Supplementary Materials), confirming the success of the impregnation procedure. It is important to note that after impregnation, the most temperature stable groups (lactone groups as well as pyrone and chromene groups) present on the parent sample RN2 evolve at higher temperatures. This could be due to the effect of the amine film on the surface of the carbon that may move the oxygen functionalities to energetically different sites.

Wet impregnation with amine causes an acidification of the carbon surface as denoted by the decrease in the CO/CO_2_ ratio. It is important to note that two different loadings of DETA (5 and 10 wt%) were tested for impregnation of the carbon RN2, and the increase in the acid character occurs in proportion to the loading employed (CO/CO_2_ ratio of 2 and 1.5 for RN2D5 and RN2D10, respectively).

The deconvoluted TPD profiles clearly show that significant chemical changes occurred during the different post-treatments. As can be observed in Table 4 heat treatment leads to a greater decrease of the carbonyls and quinones whereas in the acid washed sample an increase of 710% is observed. Moreover, RN1A is the only carbon where anhydrides are encountered. Amine impregnation reduces the number of phenols and a complete removal is attained for an amine loading of 10 wt%. Nevertheless carbonyls and quinones are completely removed for the lower loading of amine (RN2D5) and partially eliminated (50%) in RN2D10.

In Table 5 it is shown that heat treatment reduces the number of peroxides in RN1 being completely removed after acid washing. On the other hand, both post-treatments lead to an increase in the content of carboxylic groups with respect to the parent carbon RN1. It is worth noting that RN1A shows an increment in lactones of 838%. On the contrary, wet impregnation with amines decreases the content of carboxylic acids and lactones for RN2D5. However, the content of lactones is practically unchanged in the carbon with the higher loading of amine (RN2D10).

The FTIR spectra of the carbons are shown in Figure S5 in the Supplementary Materials. Heat treatment of carbon RN1 causes a decrease in the relative absorbance throughout the infrared spectral bands. The FTIR spectrum of RN1P shows similar characteristic peaks to that of RN1 and confirms that heat treatment under an inert atmosphere diminishes the content of oxygen surface groups, especially those strongly acidic.

On the other hand, acid treatment of RN1 causes the opposite effect: an increase in the relative intensity of the bands as discussed above. The RN1A spectrum shows better defined and more intense characteristic peaks which suggest a higher concentration of oxygen functional groups.

The spectra of the two RN2 impregnated carbons are similar to the parent carbon. The presence of the amine coating on the carbonaceous support is evidenced by a small peak at around 1660 cm^−1^ and the shoulder in the 3100–3400 cm^−1^ region; however, the presence of hydroxyl groups with similar vibration in the 3100–3400 cm^−1^ region make difficult a clear identification of each contribution [38]. It is also important to note that the intensity of these bands increases with the amine loading.

The FTIR results obtained for the studied samples are consistent with the TPD analysis. Post-treatments clearly influence the type and relative abundance of the oxygen functionalities present in the parent carbons.

### 3.2. Water Vapor Adsorption Isotherms

**Figure 2:**
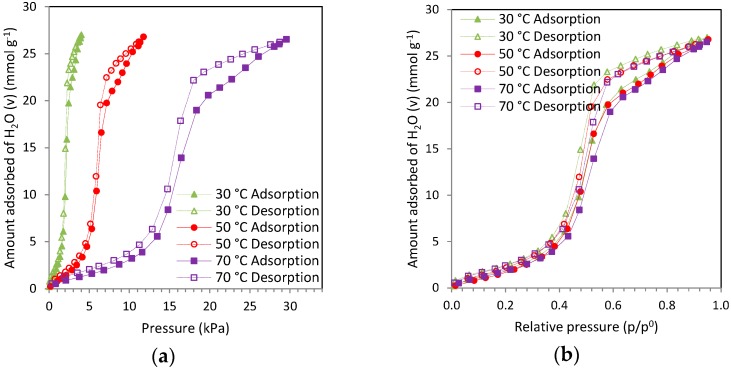
Water vapor adsorption isotherms over carbon RN2 at 30, 50, and 70 °C (full symbols and continuous lines correspond to the adsorption branch, and empty symbols and dashed lines to the desorption branch): (**a**) adsorbed amount *versus* absolute pressure; (**b**) adsorbed amount *versus* relative pressure.

Water vapor adsorption isotherms for carbon RN2 are shown in Figure 2. These isotherms present a type V topology, according to the IUPAC classification, characterized by little water uptake at low relative pressures and the presence of a hysteresis loop over the majority of the pressure range. Adsorption data have been plotted in terms of absolute pressure (a) and relative pressure (b) for illustrative purposes. Temperature limits the pressure range for the adsorption of water vapor due to condensation conditions (see Figure 2a). For instance, the vapor pressure (*p*^0^) of water at 30 °C is 4.24 kPa whereas at 70 °C it shifts up to 31.18 kPa.

As can be seen from Figure 2b, the adsorption branch is little influenced by temperature: the isotherms at 30 and 50 °C overlap in the entire relative pressure range, and at 70 °C the uptake is similar up to a relative pressure of 0.5. From this point the adsorbed amount slightly decreases, as expected for adsorption processes. The microporous nature of carbon RN2 may account for this behavior: water vapor adsorption in ultramicropores is considered nearly independent of temperature [39].

**Figure 3:**
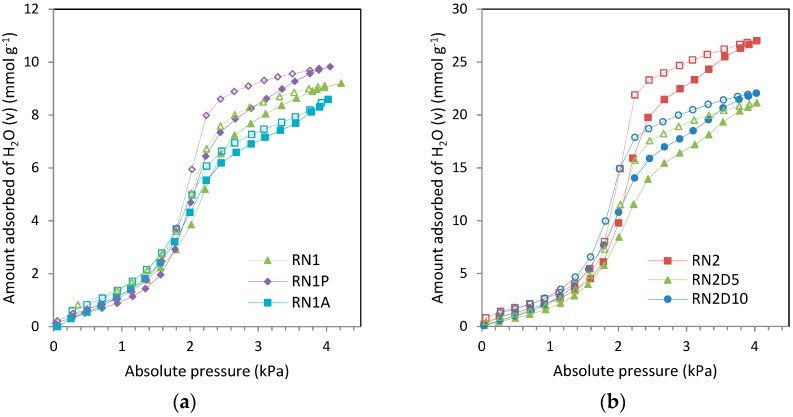
Water vapor adsorption isotherms at 30 °C: (**a**) RN1, RN1P, and RN1A; (**b**) RN2, RN2D5, and RN2D10 (full symbols and continuous lines correspond to the adsorption branch, and empty symbols and dashed lines to the desorption branch).

Heat treatment of carbon RN1 (sample RN1P) tends to shift both branches of the water vapor isotherm (adsorption and desorption) up to higher uptakes due to the previously discussed widening of the porosity. However, it decreases the amount of water vapor adsorption below *p*/*p*^0^ = 0.2; this is attributed to a decrease in oxygen surface groups (see Figure 3a).

RN1A exhibits opposite performance to RN1P in the higher pressure range (*p*/*p*^0^ > 0.5): the water vapor uptake is notably reduced with respect to RN1 due to blocking of some microporosity by chloride groups [14]. Despite the increase in the total oxygen content of RN1A, the adsorption capacity in the region below *p*/*p*^0^ = 0.2 is slightly lower than that of RN1; this might suggest that not all of the oxygen content of sample RN1A is available for H_2_O adsorption (see Figure 3a).

Regarding the impregnated carbons (see Figure 3b) the loading of amine seems to notably influence the water vapor adsorption performance. For instance, in the lower pressure range (*p*/*p*^0^ < 0.3) the isotherms of RN2 and RN2D10 nearly overlap whereas the adsorption capacity of RN2D5 is slightly reduced. On the other hand, in the higher pressure range the blocking of the porosity due to the amine film significantly reduces the water vapor uptake, this reduction being more pronounced in the sample with a lower loading of amine RN2D5. The reported positive effect of the amine in terms of water vapor adsorption [40] is only evident at the higher loading (10 wt%) where a compensation of the negative effect of the partial blocking of the porosity is observed [26].

### 3.3. Semi-Empirical and Theoretical Models for Water Vapor Adsorption

The sorption of water in the pores of activated carbons is known to be mediated by surface chemistry. Several semi-empirical and theoretical water adsorption models incorporating the role of surface chemistry can be found in the literature. Typically these models can predict the concentration of surface functional groups, the molecular size of water clusters, and the water adsorption capacity in the micropores as well as on the surface functional groups, and also estimate equilibrium rate constants. Several of these models such as the Dubinin-Serpinsky (DS) equation [12], the Dubinin-Astakhov (DA) equation [13], and the Do-Jumpirom-Do (DJD) equation [14] were used in this work to describe water vapor adsorption on the carbons at 30 °C.

#### 3.3.1. Dubinin-Serpinsky (DS)

Water adsorption isotherms have been fitted to the Dubinin-Serpinsky (DS) model [12] (see Supplementary Materials for clarification on the notation of Equation (1)).

(1) $$a\;p^{0}/p=A_{1}+A_{2}a-A_{3}a^{2}$$

The experimental data were fitted over a suitable range of *p/p*^0^, usually from 0.3 to 0.6. The parameters *A*_1_, *A*_2_, *and A*_3_ of the DS model were adjusted to minimize the sum of square residuals between the experimental adsorption data and the values calculated using DS at the adsorption temperature. As an example, the experimental data and model approximation for RN2, RN2D5, and RN2D10 carbons are shown in Figure S6 in the Supplementary Materials.

Each sample exhibits a region where the quadratic fitting of the DS equation applies and so the parameter *a*_0_ for the carbons studied can be estimated.

Table 6 reports the DS parameter values estimated for the carbons. The model fits reasonably well the experimental data up to intermediate pressures but it should be taken into account that in general, the DS equation is restricted to describe the initial region of the water vapor isotherm (0 < *p/p*^0^ < 0.3).

The number of primary centers, represented by *a*_0_, decreases after all the post-treatments, either because of the loss of oxygen surface groups due to heat treatment or due to the unavailability of these groups for water adsorption owing to the acid wash or wet impregnation procedures.

This trend is consistent with the TPD and FTIR analysis for several samples. Nevertheless RN1A shows a lower content of oxygen surface functional groups than that obtained from these techniques. As we have explained above, this clearly suggests that not all of the oxygen content of the samples is available for H_2_O adsorption (see Figure 3).

As suggested by Dubinin, *a*_0.6_ represents the amount of water adsorbed near *p/p*^0^= 0.6 and corresponds in many cases to the monolayer covering the walls of the micropores. Since *a*_0.6_ is close to the true surface of these pores (not to be confused with the total micropore volume W_0_), the ratio *a*_0_/*a*_0.6_ represents the fraction of the surface occupied by the primary centers. This ratio, rather than *a*_0_ alone, is a useful parameter for the characterization and comparison of different activated carbons [41].

It is noteworthy that for the impregnated samples the ratio *a*_0_/*a*_0.6_ remains constant with respect to the parent sample. Thus the reduction of the number of oxygen functional groups available to water vapor adsorption and the total adsorption capacity decrease in the same way independently of the amine loading for the impregnation, maintaining the proportion of the parent sample.

The DS equation might not be the most suitable model for a detailed description of water adsorption in microporous carbon adsorbents over the whole relative pressure range. Nevertheless, the DS equation showed a fair description of the adsorption of water vapor at low relative pressures thus providing information about the surface oxygen functionalities on the carbons.

#### 3.3.2. Dubinin-Astakhov (DA)

Water adsorption isotherms were fitted to the Dubinin-Astakhov (DA) equation [13].

(2) $$N_{a}=N_{a0}\;exp\left[-{\left(A/E\right)}^{n}\right]$$

The experimental data were fitted over a suitable range of *p/p*^0^; usually from 0.4 to 0.95. The optimum value of *n* for the DA equation was calculated by linear regression and selected as the value of *n* that minimizes the residual sum of squares.

As an example, the experimental data and model approximation for RN2, RN2D5, and RN2D10 carbons are shown in Figure S7 in the Supplementary Materials. The model fits reasonably well the experimental data in the pressure range evaluated. Table 7 reports the estimated DA parameters for all the studied carbons.

Estimation of the micropore volume by means of water vapor adsorption together with the liquid water density generally yields lower values than when estimated from other gaseous adsorbates (e.g., nitrogen or carbon dioxide). In such a confined space, molecular packing is not as effective as bulk liquid water. Iiyama *et al.* showed by an X-ray diffraction technique in a slit-shaped carbon nanospace that the adsorbed water has a more ordered structure. This is thought to be an ice-like structure, with a lower density than liquid water [42].

In spite of that, the micropore volume obtained for RN1P was higher (0.17 cm^−3^·g^−1^) than that calculated from nitrogen adsorption (0.11 cm^−3^·g^−1^). Depending on the pore shape and size, the packing of water molecules may not be as effective as that of gases because of the requirement of a correct orientation for hydrogen bonding. As a result, water molecules occupy only a fraction of the micropore volume while gases, such as argon, can occupy effectively the whole volume. In rare cases where micropores are very small, water might access due to its smaller size while argon or nitrogen could not.

Moreover although the estimated micropore volume of RN1 is lower than that reported in the textural characterization, the widening of the pores by means of the heat treatment leads to higher water adsorption on RN1P thereby obtaining an overestimated micropore volume.

In the case of the *E* parameter, the data presented in this work suggest that no direct correlation exists between *E* and the classical characteristic energy, *E*_0_, associated with type I isotherms. The highest value is registered for RN1P (3.6 kJ·mol^−1^) and may be ascribed to the volume of micropores of the sample (W_0,N2_ = 0.11). This could be explained by the fast filling of the micropores that leads to the cooperative adsorption of clusters in the form of multilayer on the carbon surface. These clusters of water act as secondary active centers by means of hydrogen bonding and increase the value of *E* due to their evident affinity. The lower values were obtained for the aminated samples (about 2–3 kJ·mol^−1^) owing to the presence of amine groups that diminish the number of oxygen surface functional groups besides blocking the entrance to the micropores.

Even though water adsorption on the carbons is of type V, the DA equation was insufficient to describe experimental data at low relative pressures. This is because the DA equation only describes the volume filling of micropores at medium and high pressures and does not take into account the presence of surface oxygen functional groups responsible of water vapor adsorption at low pressures.

#### 3.3.3. Do-Junpirom-Do (DJD)

Water adsorption isotherms were fitted to the Do-Junpirom-Do (DJD) equation [14].

(3) $$C_{T,\;adsorption}=S_{0}\frac{K_{f}\sum_{n=1}^{m}nx^{n}}{1+K_{f}\sum_{n=1}^{m}x^{n}}+C_{\mu s}\frac{K_{\mu }\sum_{n=\alpha_{\mu }+1}^{m}x^{n}}{K_{\mu }\sum_{n=\alpha_{\mu }+1}^{m}x^{n}+\sum_{n=\alpha_{\mu }+1}^{m}x^{n-\alpha_{\mu }}}$$

(4) $$C_{T,\;desorption}=S_{0}\frac{K_{f}\sum_{n=1}^{m}nx^{n}}{1+K_{f}\sum_{n=1}^{m}x^{n}}+C_{\mu s}\frac{K_{\mu }\left(1+K_{R\mu }\right)\sum_{n=\alpha_{\mu }+1}^{m}x^{n}}{K_{\mu }\left(1+K_{R\mu }\right)\sum_{n=\alpha_{\mu }+1}^{m}x^{n}+\sum_{n=\alpha_{\mu }+1}^{m}x^{n-\alpha_{\mu }}}$$

The parameters *S*_0_, *K_f_*, *α_μ_*, *C_μs_*, and *K_μ_* of the DJD model were adjusted to minimize the sum of square residuals between the experimental adsorption data and the values calculated using Equation (3) at the adsorption temperature. The portion of the isotherm corresponding to the filling of the micropores (*p/p*^0^ > 0.2) was first fitted to obtain *C_μs_*, and *K_μ_*; then, a new optimization was run departing from these preliminary adjusted parameters by fitting all the parameters simultaneously using the total sum of the square residuals of adsorption as the objective function. The value of *m* was set to *m* = *α_μ_* + *1*. The relaxation equilibrium constant for water desorption from the micropores, *K_Rμ_*, was fitted to minimize the sum of square residuals between the experimental desorption data and the values calculated using Equation (4). In order to fit the parent activated carbons, RN1 and RN2, previous results optimized by our research group were used and these values were applied as initial in the fitting of the treated samples [15,39].

Heat treatment reduces the number of oxygen surface functional groups in sample RN1P, leads to a decrease in the micropore volume of the parent carbon (see Table 2) but increases the total water vapor uptake of RN1P. Therefore the parameters *S*_0_ and *C_μs_* vary for the heat treated sample RN1P with respect to the parent RN1. Moreover *C_μs_* was set to be greater or equal to the physical upper limit (6.08 mmol·g^−1^) which is calculated assuming that all the micropore volume is completely full of liquid water at the adsorption temperature.

In the case of the acid treated sample RN1A and the aminated samples RN2D5 and RN2D10 the values of *C_μs_* optimized for the parent activated carbons were set as constants in the fitting. Although these samples present a reduction in microporosity with respect to the parent carbons, their performance in water vapor adsorption only seems to be affected in the pressure range assigned to the contribution of oxygen surface functional groups where they show a decrease in the total water vapor uptake capacity. However, the water vapor uptake corresponding to the filling of micropores remains unaltered with respect to the parent samples. Thus only the parameter *S*_0_ is considered to be affected by the effect of these post-treatments.

The values of the DJD model parameters optimized following the aforementioned criteria can be found in Table 8.

The model prediction with the experimental isotherms at the evaluated temperature for samples RN2, RN2D5, and RN2D10 are presented in Figure S8 in the Supplementary Materials.

It can be observed that the DJD model (Equation (3)) describes adequately the adsorption branch of the isotherms in the entire pressure range at the evaluated temperature. However, the DJD model for desorption (Equation (4)) presents greater deviations from the experimental desorption data: it underestimates the uptake at high pressures, particularly for RN2, which corresponds to the emptying of the widest micropores. This seems to be an intrinsic limitation of the model. Besides, only one parameter (*K_Rμ_*) was fitted using the experimental desorption data; the rest of the model parameters (*S*_0_, *K_f_*, *α_μ_*, *C_μs_*, *and K_μ_*) were fitted to minimize the sum of square residuals between the experimental adsorption data and the adsorption model (Equation (3)).

The values obtained for *S*_0_ represent between 7% and 30% of the total oxygen content of the carbons, which was to be expected, as not all of the oxygen is available for H_2_O adsorption. The critical size of the water cluster to enter the micropores, *α_μ_*, is related to the micropore size and also varies with the concentration of functional groups; when their amount is large the water clusters are small and *vice versa*. This is due to the functional groups that can contribute to stabilize smaller clusters, allowing their confinement within the micropores [14].

Regarding the parent activated carbons, the value of *α* = 6 obtained for RN1 is relatively low, which is attributed to the narrow size of the micropores of this carbon. In fact, this value is lower than that obtained for RN2 which is produced from the same precursor by activation with CO_2_ and presents slightly higher oxygen content but wider micropores (see Table 2).

For parent carbon RN1, *C_μs_* represents 57% of the physical upper limit (11.06 mmol·g^−1^). This packing fraction in the micropores is also significantly lower for RN1 than for RN2 (74%), due to the narrower pore size of the carbon activated in air.

Despite that the size of the water cluster for the samples with low content of oxygen surface functional groups should be larger than for the parent carbon, in the case of RN1P the value of *α* remains constant. For this carbon, the value of *C_μs_* represents 100% of the physical upper limit, due to its poor microporosity development as previously commented within the DA section.

Acid treatment led to a decrease in the size of the water cluster (carbon RN1A) due to the presence of remaining chloride groups that could contribute to stabilize smaller clusters. The number of primary centers represented by *S*_0_ also decreases because of the unavailability of these groups for water vapor adsorption after this treatment.

Likewise, impregnation with amines followed the same trend as acid treatment: decrease in the size of the cluster due to the presence of amine that acts as oxygen surface functional group and stabilizes the clusters inside the pores. Moreover S_0_ decreases owing to the effect of the wet impregnation.

In order to describe the water vapor adsorption performance of the studied samples, the main advantages of the DJD model are that it provides a fair description of the adsorption branch in the entire relative pressure range and that it is based on the specific mechanism of water vapor adsorption on carbon materials.

### 3.4. Adsorption Equilibrium of Pure CO_2_ and N_2_

**Figure 4:**
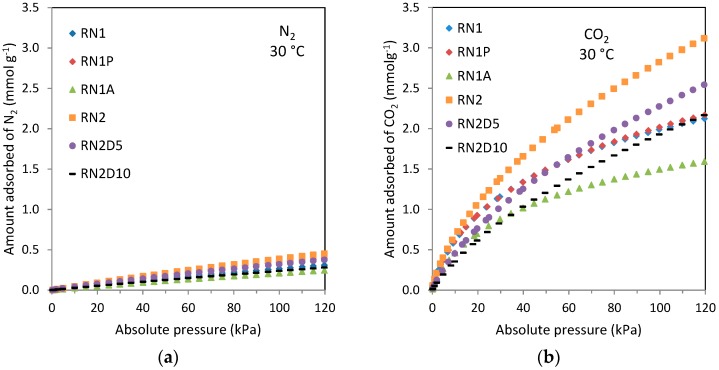
Equilibrium adsorption isotherms of: (**a**) N_2_ and (**b**) CO_2_ at 30 °C of carbons RN1, RN1P, RN1A, RN2, RN2D5, and RN2D10.

The capacity of the carbons to adsorb CO_2_ and N_2,_ main components of flue gas streams, was also evaluated. The CO_2_ and N_2_ adsorption isotherms of the carbons at 30 °C are presented in Figure 4. All the carbons present greater CO_2_ adsorption capacity than N_2_ over the entire pressure range. Globally, the N_2_ isotherms exhibit linear patterns (Henry-type), characteristic of weak adsorbate-adsorbent interaction forces, whereas the CO_2_ adsorption isotherms can be classified as Type I, *i.e.*, they are representative of stronger adsorbate-adsorbent interactions. This difference can be attributed to the larger quadrupole moment of the CO_2_ molecule (about three times that of the N_2_ molecule), due to the strong dipole moment of carbonyl bonds [43]. Thus, carbons show greater selectivity towards CO_2_.

In contrast to the parent carbon, RN2, the aminated samples RN2D5 and RN2D10 present lower CO_2_ adsorption capacity over the entire pressure range evaluated. This is mainly due to the contribution of physisorption, which is limited in the case of the modified sorbents owing to the pores being blocked by the amine film. Likewise the activated carbon RN1 presents higher CO_2_ adsorption capacity than the acid washed sample RN1A because of the blocking of the pores by the chloride groups as previously commented. However, heat treatment (RN1P) seems to little influence the CO_2_ uptake at 30 °C over the entire pressure range. The widening of the microporosity, as reported in Table 2, even favors a slight increase in the CO_2_ uptake of carbon RN1P with respect to RN1 at pressures close to atmospheric.

It is also worth noting that despite their differences in total pore volume, carbons RN1, RN1P, and RN2 show similar CO_2_ adsorption capacities (0.78 mmol·g^−1^) in the lower pressure range (*p* < 15 kPa), conditions particularly relevant for post-combustion CO_2_ capture purposes. Regarding N_2_ adsorption, there are no significant differences in uptake between the carbons given the unspecific nature of the interaction between N_2_ and the carbon surface.

## 4. Conclusions

This study evaluated the changes in surface chemistry and porous texture of biomass-based carbons subjected to different post-treatments and how these impacted their water vapor adsorption performance.

Heat treatment removed most of the oxygen on the carbon surface whereas wet impregnation with amines and acid washing increased it. Similar oxygen surface functionalities were identified in all the carbons by means of TPD tests and FTIR. Moreover impregnation with amines successfully incorporated nitrogen functionalities by reaction with oxygen moieties of the carbon support. On the other hand, all evaluated post-treatments led to a decrease in surface area and micropore volume.

Independently of the parent carbon (RN1 or RN2) the effect of all post-treatments was a decrease of water vapor adsorption in the first part of the isotherm (*p*/*p*^0^ < 0.2). In the higher pressure ranges, post-treatments also reduced the total water vapor uptake with the exception of heat treatment that led to an increase of 6.8% with respect to the parent carbon. Wet impregnation seems to be the most effective to attain an important reduction in the water vapor adsorption capacity.

The DS equation fitted reasonably well the experimental data in the initial region of the isotherm (0 < *p/p*^0^ < 0.3). Therefore it may be a suitable model to assess the contribution of surface oxygen functional groups in the samples. On the other hand, the DA equation satisfactorily describes water vapor adsorption at intermediate pressures governed by the volume filling of micropores. Thus, this equation may also address the micropore volumes from water vapor adsorption data. The Do-Junpirom-Do (DJD) model provides a fair description of water vapor adsorption within the entire pressure range, although it shows certain limitations to reproduce the desorption branch.

It is well known that flue gas from coal-fired power plants contains roughly about 73%–77% N_2_, 15%–16% CO_2_, 5%–7% H_2_O, 3%–4% O_2_ and trace amounts of impurities such as SO_2_ and NOx. Therefore post-combustion flue gas may be saturated with water vapor. In this context, only the Do-Junpirom-Do (DJD) model may adequately describe the adsorption of water vapor. Regarding the performance of the studied biomass based carbons to capture CO_2_ under these humid post-combustion capture conditions, acid washing of biomass based carbons can be seen as a potential alternative. The CO_2_ uptake at 15 kPa (representative of a coal fired power plant) is slightly reduced with respect to the parent carbon but the H_2_O uptake at pressures close to saturation also noticeably decreases. Nevertheless wet impregnation showed a substantial reduction of water vapor adsorption. There is a trade-off between the reduction of the water uptake and the enhancement of the CO_2_ uptake at pressures relevant to post-combustion capture.

## Figures and Tables

**Table 1:** Chemical analysis of the samples.

Sample	Proximate Analysis (wt%)		Ultimate Analysis (wt%, daf ^1^)			
	Ash	Humidity	C	H	N	O
RN1	1.8	4.6	90.8	1.4	0.4	7.4
RN1P	0.4	5.9	94.6	0.7	0.6	4.2
RN1A	0.0	3.2	88.1	1.6	0.4	9.9
RN2	6.3	10.4	87.1	0.9	0.7	11.3
RN2D5	5.2	9.9	85.6	1.4	1.3	11.6
RN2D10	4.4	3.6	81.2	1.7	3.8	13.4

^1^ dry ash free basis.

**Table 2:** Textural parameters of the samples obtained from the N_2_ and CO_2_ adsorption isotherms.

Sample	dHe (g·cm^3^)	N_2_ Adsorption (−196 °C)						CO_2_ Adsorption (0 °C)				
		Vp ^1^	S_BET_^2^	W_0_^1^	E_0_^3^	L_0_^4^	S_mi_^2^	*n*	W_0_^1^	E_0_^3^	L_0_^4^	S_mi_^2^
RN1	1.80	0.21	514	0.20	25.36	0.77	516	2	0.20	33.03	0.50	798
RN1P	2.01	0.13	281	0.11	21.02	1.12	195	2	0.22	30.52	0.56	796
RN1A	1.73	0.15	348	0.14	19.12	1.40	199	2	0.19	32.43	0.51	742
RN2	2.10	0.53	1248	0.48	21.32	1.09	888	1.7	0.44	24.94	0.80	1112
RN2D5	1.86	0.47	1035	0.41	21.03	1.12	727	2	0.30	27.54	0.67	910
RN2D10	1.82	0.46	1014	0.40	20.37	1.20	664	2	0.28	26.46	0.72	784

^1^ V, W [=] cm^3^·g^−1^; ^2^ S [=] m^2^·g^−1^; ^3^ E_0_ [=] kJ·mol^−1^; ^4^ L_0_ [=] nm.

**Table 3:** Amount of CO and CO_2_ evolved during the temperature programmed desorption (TPD) experiments.

Sample	CO (µmol·g^−1^)	CO_2_ (µmol·g^−1^)	CO/CO_2_	CO + CO_2_ (µmol·g^−1^)
RN1	1438	884	1.6	2322
RN1P	824	308	2.7	1131
RN1A	3176	2645	1.2	5821
RN2	2249	890	2.5	3139
RN2D5	943	473	2.0	1416
RN2D10	840	577	1.5	1417

**Table 4:** Oxygen surface complexes distribution from CO TPD profiles.

Sample	CO (μmol·g^−1^)			
	Anhydride	Phenol	Carbonyl and Quinone	Pyrone and Chromene
RN1	–	–	380	620
RN1P	–	–	14	140
RN1A	137		1310	–
RN2	–	52	60	800
RN2D5	–	32	–	250
RN2D10	–	–	30	270

**Table 5:** Oxygen surface complexes distribution from CO_2_ TPD profiles.

Sample	CO_2_ (μmol·g^−1^)			
	Carboxylic	Anhydride	Peroxide	Lactone
RN1	100	–	470	300
RN1P	130	–	40	130
RN1A	250	20	–	1850
RN2	400	–	–	430
RN2D5	210	–	–	220
RN2D10	100	–	–	420

**Table 6:** Dubinin-Serpinsky (DS) parameters estimated from water adsorption at 30 °C on the studied carbons. The amounts of a_s_, a_0.6_, and a_0_ are given in mmol·g^−1^.

Sample	c	a_0_	k	a_s (0.95)_	a_0.6_	a_0_/a_0.6_	(a_0_/a_s_) 10^2^	R^2^
RN1	1.77	1.50	0.034	9.09	6.87	0.22	16.5	0.999
RN1P	2.06	0.79	0.027	9.83	7.58	0.10	8.1	1.000
RN1A	2.30	0.90	0.055	8.59	6.35	0.14	10.5	0.999
RN2	1.86	2.45	0.008	27.00	20.54	0.12	9.1	0.999
RN2D5	2.14	1.51	0.019	21.15	14.64	0.10	7.1	1.000
RN2D10	2.26	1.76	0.018	22.04	16.32	0.11	8.0	1.000

**Table 7:** Parameters estimated from the fitting of the H_2_O adsorption isotherms to the Dubinin-Astakhov (DA) equation.

Sample	H_2_O Adsorption at 30 °C				
	N_a0_ (mmol·g^−1^)	W_0_ (cm^3^·g^−1^)	E (kJ·mol^−1^)	*n*	R^2^
RN1	8.97	0.16	2.48	2.56	0.994
RN1P	9.36	0.17	3.55	3.33	0.991
RN1A	8.09	0.15	3.03	2.39	0.985
RN2	25.80	0.47	2.85	3.37	0.990
RN2D5	20.12	0.36	2.25	2.48	0.990
RN2D10	21.18	0.38	2.83	2.47	0.987

**Table 8:** Do-Junpirom-Do (DJD) parameters for the water vapor adsorption isotherms at 30 °C of the studied carbons.

Sample	*S*_0_ (mmol·g^−1^)	*K_f_*	*α_μ_*	*C_µs_* (mmol·g^−1^)	*K_μ_*	*K_Rμ_*	R^2^
RN1	0.80	33.88	6	6.30	69	1.08	0.997
RN1P	0.55	46.39	6	7.81	82	0.84	0.997
RN1A	0.66	25.69	5	6.30	45	0.43	0.996
RN2	1.62	29.27	8	19.54	281	1.32	0.997
RN2D5	0.63	116.43	5	19.54	26	0.91	0.998
RN2D10	0.82	102.55	5	19.54	38	0.92	0.998

## Supplementary Materials

The following are available online at www.mdpi.com/1996-1944/9/5/359/s1.

## Abbreviations

The following abbreviations are used in this manuscript:

CCS	Carbon capture and storage
MOFs	Metal organic frameworks
GCMC	Grand Canonical Monte Carlo method
ACs	Activated carbons
DS	Dubinin-Serpinsky
DA	Dubinin-Astakhov
DJD	Do-Junpirom-Do
OS	Olive stones
DETA	Diethylenetriamine
BET	Brunauer-Emmett-Teller
TPD	Temperature Programmed Desorption
FTIR	Fourier Transform Infrared Spectroscopy
