# Supplementary material for: Water Vapor Adsorption on Biomass Based Carbons under Post-Combustion CO2 Capture Conditions: Effect of Post-Treatment
Source: Materials (Basel). 2016 May 12;9(5):359. doi: 10.3390/ma9050359 (PMC5503013; doi:10.3390/ma9050359)
Supplement: Supplementary file 1 [file materials-09-00359-s001.pdf]

Supplementary Materials: Water Vapor Adsorption on Biomass Based Carbons under Post-Combustion CO_2 Capture Conditions: Effect of Post-Treatment

Nausika Querejeta, Marta G. Plaza, Fernando Rubiera and Covadonga Pevida

1. Methods for the Modification of Activated Carbon

Impregnation with Amines

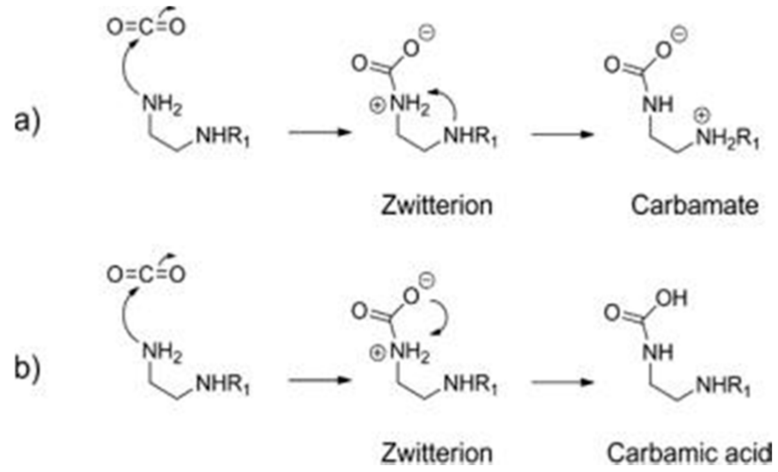

Figure S1. CO_2 adsorption on bifunctional amines (primary and secondary amine groups) via: (**a**) the carbamate mechanism and (**b**) the formation of carbamic acid.

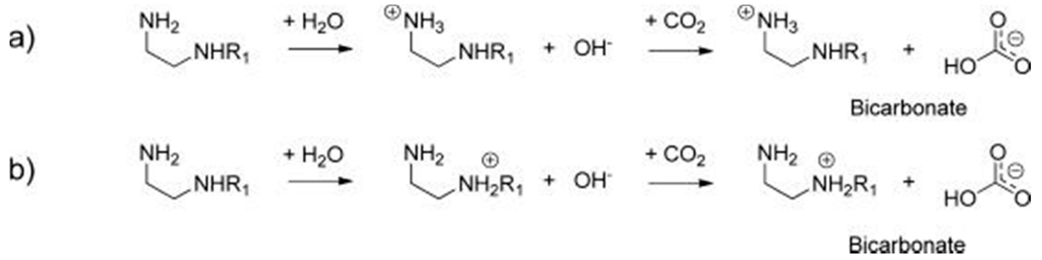

Figure S2. CO_2 adsorption on bifunctional amines by formation of bicarbonates via: (**a**) primary and (**b**) secondary amine groups.

2. Characterization of the Samples

2.1. Temperature Programmed Desorption (TPD)

Temperature programmed desorption (TPD) tests were carried out in a thermogravimetric analyzer, Setaram TGA92, coupled to an Omnistar™ mass spectrometer from Pfeiffer Vacuum. Around 20 mg of carbon sample were placed in a platinum crucible (100 μL) and heated from ambient temperature to 1000 °C (heating rate of 15 $°C \cdot min^{-1}$) under flowing Argon (50 $cm^3 \cdot min^{-1}$). Prior to these measurements calibration tests with calcium oxalate were carried out. It is well known that upon heating in inert atmosphere, the oxygen surface complexes of carbonaceous materials decompose, releasing CO_2 and CO. CO_2 results from the decomposition of carboxyls, lactones and anhydrides, while CO comes from anhydrides, phenols, carbonyls, quinones, and pyrones. Mass to charge (m/z) values of 18, 28, and 44 were monitored with the mass spectrometer to account for the evolution of H_2O, CO and CO_2, respectively. Scientific data analysis and graphing software was used to fit the TPD curves with GaussianAmp peaks. There was no baseline subtraction.

Figure S3 shows the TPD plots (CO_2 and CO evolution) for the initial carbons and the samples subjected to post-treatment.

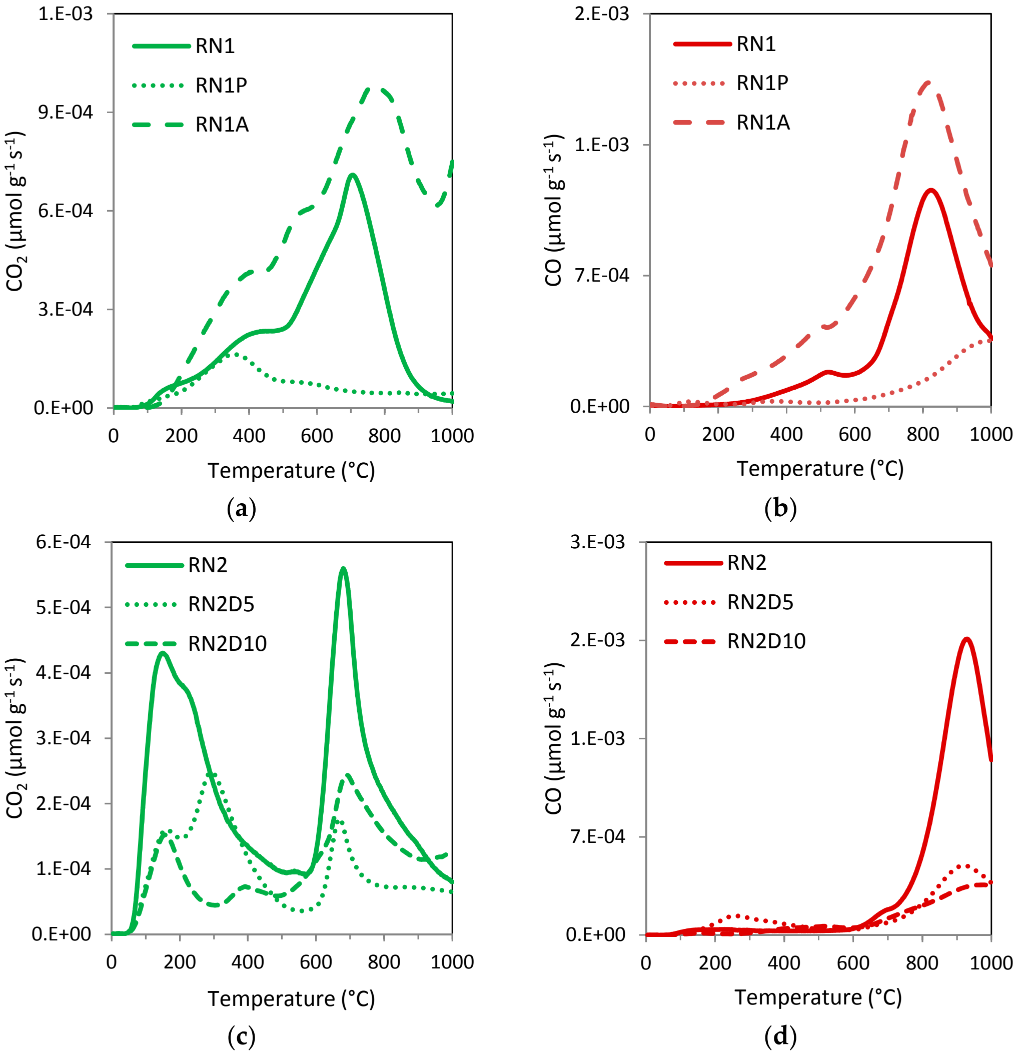

Figure S3. Temperature programmed desorption (TPD) profiles for the studied carbons: (**a**) CO_2 evolution of RN1, RN1P, and RN1A; (**b**) CO_2 evolution of RN2, RN2D5, and RN2D10; (**c**) CO evolution of RN1, RN1P, and RN1A; (**d**) CO evolution of RN2, RN2D5, and RN2D10.

For sample RN1 the shoulder in the CO_2 profile at 600 °C indicates the presence of peroxides [1] and the main peak located at 719 °C is assigned to more stable oxygenated groups, such as lactones [2]. Moreover, at very low temperatures, around 215 °C, there is a first peak associated with strong carboxylic acid groups and a second peak at 365 °C that corresponds to less acidic carboxylic groups [1]. Maximum CO-desorption takes place at around 820 °C and is ascribed to the evolution of carbonyls and quinones; at 973 °C CO desorption continues due to the decomposition of pyrone and chromene groups, whose contributions are difficult to isolate [2]. Similarly, CO desorption also occurs at lower temperature, around 490 °C, and may be due to the thermal decomposition of carbonyl groups in α-substituted ketones and aldehydes [3].

The main peak in the CO_2-profile appears at 349 °C and it is associated to less acidic carboxylic groups [1]. Likewise the main peak in the CO-profile is located at 999 °C and is related with pyrone and chromene groups [2]. The appearance of small CO desorption peaks at low temperatures, 125 and 362 °C respectively, may be due to the thermal decomposition of carbonyl groups in α-substituted ketones and aldehydes [3].

Due to the acid treatment anhydrides are generated in RN1A by the reorganization of oxygen surface groups (527 °C). Anhydrides can be formed from the dehydration of two neighboring

carboxylic groups, and decompose generating CO and CO_2 simultaneously. However, it can be observed that the CO_2 peak tends to appear shortly before the CO peak. In fact, the decomposition of carboxylic anhydrides is not a simultaneous process: a CO_2 molecule desorbs in first place and is then followed by the CO one. Nevertheless, they are usually considered to be simultaneous [4]. The main peak in the CO_2-profile appears at 780 °C and it is associated to lactone groups [3]. In the CO-profile the main peak is located at 823 °C and it is ascribed to carbonyl and quinone groups [2]. In addition, the appearance of a small CO peak at low temperature (364 °C) may be due to the thermal decomposition of carbonyl groups in α-substituted ketones and aldehydes [3]. On the other hand, desorption of oxygen groups in the form of CO and CO_2 continues above 1000 °C.

The CO_2 profile of RN2 shows its main peak at 680 °C followed by a large tail with a characteristic peak at 753 °C. They are attributed to the same group located on different energetic sites [5,6]. These are assigned to lactones [2,3]. At very low temperatures, around 156 °C, there is a first peak associated with strong carboxylic acid groups and a second peak at 273 °C that corresponds to less acidic carboxylic groups [1]. The main peak of CO desorption takes place at around 929 °C, preceded by an earlier peak at 800 °C. Different CO-evolving groups, as previously commented, may account for this: pyrone and chromene groups as well as the carbonyls and quinones, respectively. Similarly, CO desorption also occurs at lower temperature, around 700 °C, being assigned to phenol groups [2]. The appearance of small CO desorption peak at low temperatures 333 °C, may be due to the thermal decomposition of carbonyl groups in α-substituted ketones and aldehydes [3].

For the RN2 samples impregnated with amines the rate of mass loss is also presented in Figure S4 since amines are known to be very labile compounds. The three first peaks in the DTG curve are due to the amine solution (T < 500 °C) whereas above 600 °C the mass loss is attributed to the carbonaceous support. More precisely, the first peak (~100 °C) is related to the evolution of physically adsorbed water at low temperatures, the second peak (~200 °C) is mostly ascribed to methanol evolution, the solvent used in the wet impregnation procedure, and, finally, the third peak (~400 °C) is attributed to NH_3 evolved from the decomposition of the immobilized amine groups [7].

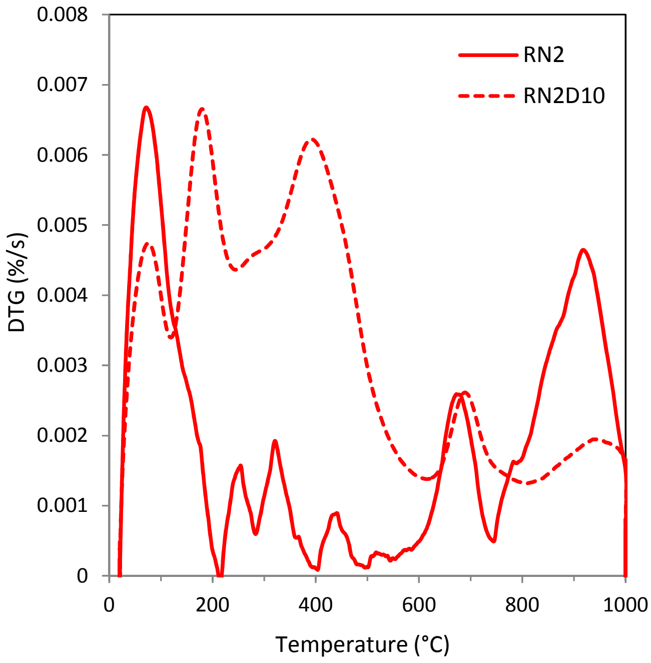

Figure S4. Thermal stability of the impregnated sample RN2D10.

For RN2D5 the main peak in the CO_2 profile may correspond to less acidic carboxylic groups that evolve at 299 °C [1] while for RN2D10 the main CO_2 peak shifted down to 698 °C followed by a large tail with a characteristic peak at 892 °C, are attributed to the same group located on different

energetic sites [5,6] corresponding to lactones [2,3]. In the CO profiles for both RN2D5 and RN2D10 the main peak is assigned to pyrone and chromene groups evolving at around 960 °C [2]. Also the appearance of two small CO desorption peaks at lower temperatures for RN2D5 and RN2D10 may be due to the thermal decomposition of carbonyl groups in α-substituted ketones and aldehydes [3].

2.2. Fourier Transform Infrared (FTIR) Spectroscopy

Infrared spectra were collected with a resolution of 4 cm^{-1} using a Nicolet 8700 Fourier Transform Infrared spectrometer with a DTGS detector. Before each measurement, the instrument was run to collect the background, which was then automatically subtracted from the sample spectrum. Discs were prepared by compressing mixtures of 0.33 wt% of finely ground carbon with KBr.

The FTIR spectra of the carbons are shown in Figure S5.

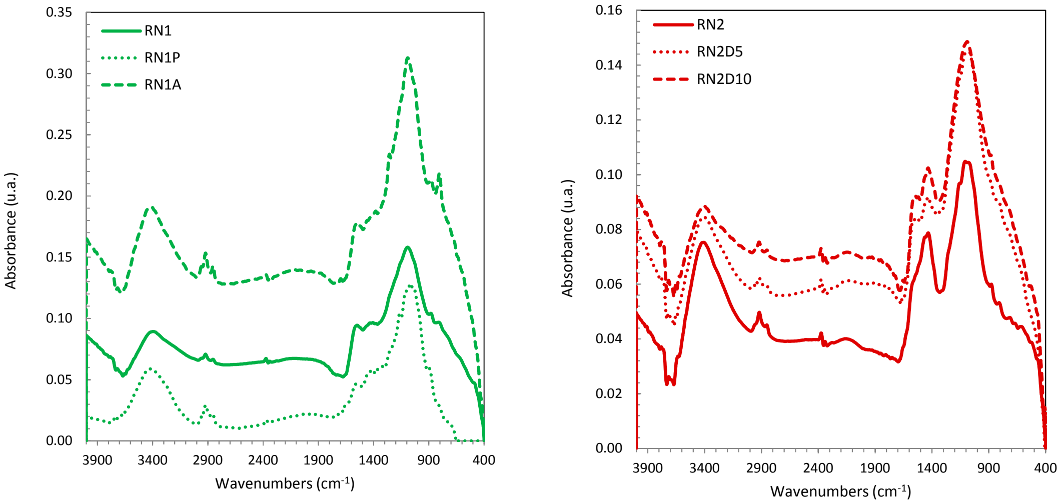

(**a**) (**b**)

Figure S5. FTIR spectra of the samples studied: (**a**) RN1 and (**b**) RN2 carbons.

The peak at around 3400 cm^{-1} might be attributed to the presence of -OH groups of water molecules in the KBr or to moisture within the measurement chamber; nevertheless, it may also be an indicative of the stretching vibrations of -OH of the carboxylic groups (COOH) [8]. Twin peaks at 2860 and 2920 cm^{-1} are assigned to the asymmetric and symmetric stretching vibrations of C-H in CH_2 groups [8–10]. A peak near 1550 cm^{-1} has been identified as due to the stretching vibrations of aromatic C=C bonds which are polarized by oxygen atoms bound near one of the C atoms [11]. In the range 1000–1450 cm^{-1}, absorbance is a consequence of the presence of various forms of oxygen surface groups such as -OH groups in phenol and carboxyl structures, C-O-C vibrations in ether structures, and thermally stable carboxyl-carbonate structures [12]. Thus, the peak in the 1180–1000 cm^{-1} region can be attributed to the presence of C-O-C groups whereas the peak at 1430 cm^{-1} may be due to the presence of CH_3 [8].

For the amine impregnated carbons the increase in the spectral bands corresponding to the peak at 1085–1100 cm^{-1} and 1560–1616 cm^{-1} can be attributed to C-N stretching vibrations and N-H bending, respectively, which supports the presence of primary and secondary amine groups. Additionally the increase in the bands at 1280 cm^{-1} and at 779–800 cm^{-1}may be related to C-N stretching vibration and to NH wagging vibration, respectively [13].

3. Semi-Empirical and Theoretical Models for Water Vapor Adsorption

3.1. Dubinin-Serpinsky (DS)

It is widely accepted that water adsorption on activated carbons takes place in three different steps: (i) chemisorption of water molecules around adsorption centers, functional groups containing

oxygen [14,15]; (ii) growth of clusters around the chemisorbed water molecules by means of hydrogen bonding; and (iii) micropore filling when the water clusters have reached a critical size and have enough dispersive energy to enter the micropores [15]. Once the water cluster has entered the micropore, it leaves a free active center for adsorption of new water molecules, and adsorption continues until the porous structure is completely filled and the active centers are surrounded by water molecules forming water clusters. One of the early attempts to explain water adsorption isotherms was made by Dubinin and Serpinsky [14]. According to their statements, the main cause for water adsorption is the primary adsorption centers and the further hydrogen bond formation with other water molecules. The Dubinin-Serpinsky (DS) [16] equation takes the following form:

$$p/p^0 = \frac{a}{c(a_0 + a)(1 - ka)} \quad (1)$$

where a is the adsorption at p/p^0; a_0 is the adsorption on primary centers; k is a constant which takes into account the decrease in active adsorption centers with increasing adsorption; and c is a kinetic constant related to the ratio of the adsorption and desorption rate constants. In fact, following the Dubinin approach, the isotherm is expressed in the form:

$$a\,p^0/p = A_1 + A_2 a - A_3 a^2 \quad (2)$$

The isotherm parameters are estimated by simultaneous solution of the identities as follows:

$$A_1 = ca_0,\ A_2 = c(1 - a_0 k),\ A_3 = ck \quad (3)$$

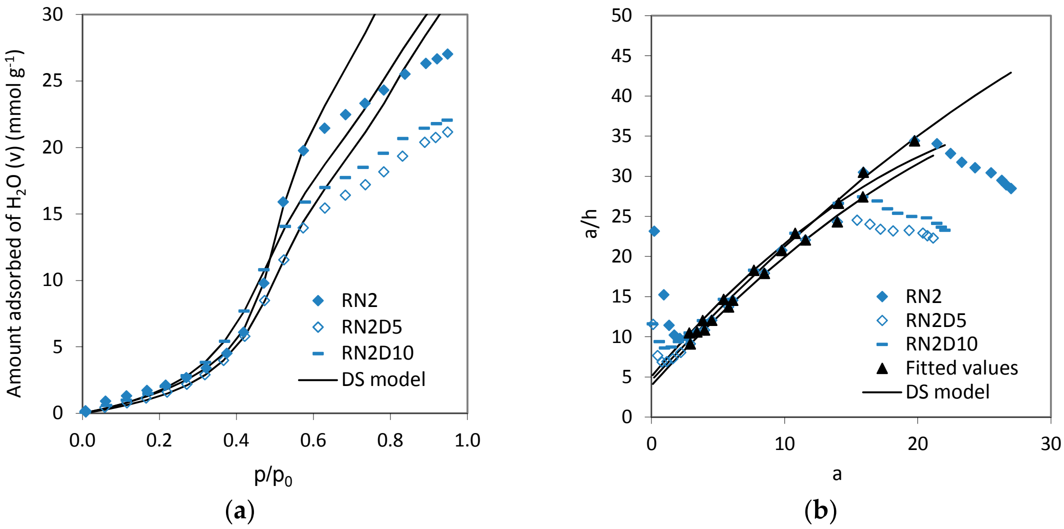

Figure S6. Dubinin-Serpinsky fitting of water adsorption data at 30 °C for carbons RN2, RN2D5 and RN2D10: (**a**) experimental and fitted water vapor isotherms; (**b**) DS equation fit, where h = p/p^0.

3.2. Dubinin Astakhov (DA)

Stoeckli *et al.* showed that the adsorption branch of the water isotherm in microporous carbons, usually of type V, could also be described by the classical equation of Dubinin and Astakhov [17].

$$N_a = N_{a0}\,exp[-(A/E)^n] \quad (4)$$

In this expression, N_a, represents the amount of water adsorbed in mmol·g^{-1} at temperature T and relative pressure p/p^0; N_{a0} is the limiting amount adsorbed in the micropores, $A = RT\,ln(p^0/p)$ and n and E are characteristics of the system under investigation. The low values of E observed for water (typically between 0.8 and 2–3 kJ·mol^{-1}) are responsible for the change from the classical type I isotherm, observed for organic vapors, to the *S*-shaped water isotherm.

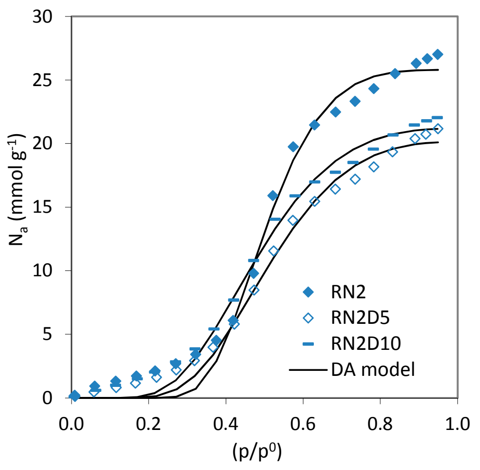

Figure S7. Dubinin-Astakhov fitting of water adsorption isotherms at 30 °C for carbons RN2, RN2D5 and RN2D10.

3.3. Do-Junpirom-Do (DJD)

One of the most complete models to describe the adsorption of H_2O on carbon materials is the Horikawa-Do model [18] propounded in 2011, which is an extension of previous adsorption models by *Do et al* [15]. This model attempts to overcome the limitations of previous models by describing the adsorption and desorption of water vapor in the entire relative pressure range. Due to the highly microporous character and the absence of mesoporosity in the carbons under evaluation the Horikawa-Do model can be reduced to the model proposed by Do, Junpirom and Do (DJD) in 2009 [19] as follows:

$$C_{T,adsorption} = S_0 \frac{K_f \sum_{n=1}^{m} n x^n}{1 + K_f \sum_{n=1}^{m} x^n} + C_{\mu s} \frac{K_\mu \sum_{n=\alpha_\mu+1}^{m} x^n}{K_\mu \sum_{n=\alpha_\mu+1}^{m} x^n + \sum_{n=\alpha_\mu+1}^{m} x^{n-\alpha_\mu}} \quad (5)$$

$$C_{T,desorption} = S_0 \frac{K_f \sum_{n=1}^{m} n x^n}{1 + K_f \sum_{n=1}^{m} x^n} + C_{\mu s} \frac{K_\mu (1 + K_{R\mu}) \sum_{n=\alpha_\mu+1}^{m} x^n}{K_\mu (1 + K_{R\mu}) \sum_{n=\alpha_\mu+1}^{m} x^n + \sum_{n=\alpha_\mu+1}^{m} x^{n-\alpha_\mu}} \quad (6)$$

where $C_{T,adsorption}$ is the total amount of H_2O adsorbed at a relative pressure x corresponding to the adsorption branch, S_0 is the concentration of the functional groups on the carbon surface, K_f is the chemisorption equilibrium constant ($K_f \approx K/K_p$, where K is the equilibrium constant for adsorption and desorption per unit functional group and K_p is the equilibrium constant for adsorption and desorption of water which adsorbs on the functional group), m represents the maximum number of water molecules that could form around one single functional group, $C_{\mu s}$ is the saturation concentration in the micropores, K_μ is the micropore equilibrium constant, α_μ represents the critical size of the water cluster to enter the micropores, $C_{T,desorption}$ is the total amount of H_2O that remains adsorbed at a relative pressure x corresponding to the desorption branch, and $K_{R\mu}$ is the relaxation equilibrium constant for water desorption from the micropores.

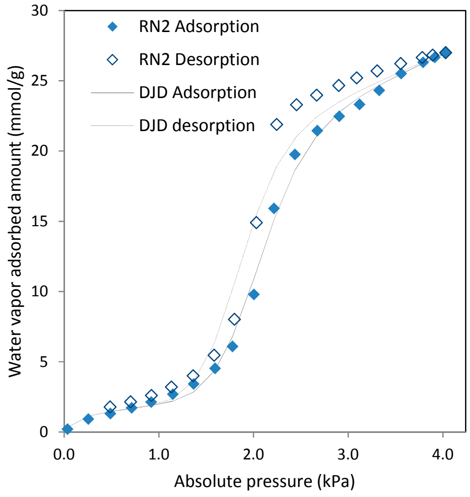

(**a**)

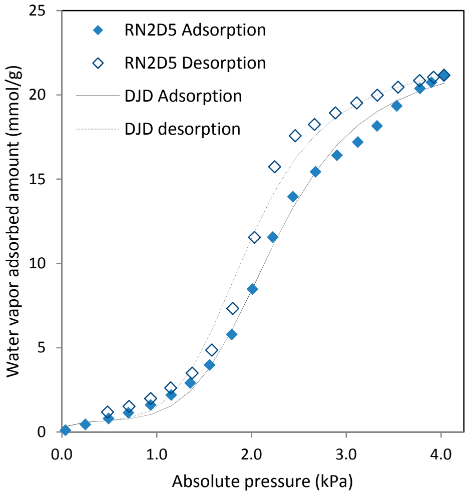

(**b**)

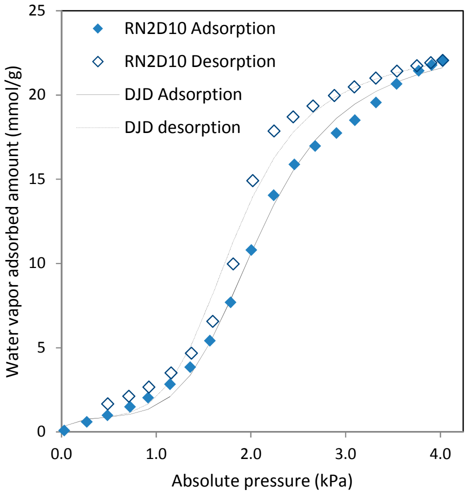

(**c**)

Figure S8. Goodness of fit of the Do-Junpirom-Do (DJD) model to the experimental adsorption isotherms of water vapor on: (**a**) RN2; (**b**) RN2D5, and (**c**) RN2D10.

References

1. Zielke, U.; Hüttinger, K.J.; Hoffman, W.P. Surface-oxidized carbon fibers: I. Surface structure and chemistry. *Carbon* **1996**, *34*, 983–998.
2. Figueiredo, J.; Pereira, M.F.; Freitas, M.M.; Órfão, J.J. Modification of the surface chemistry of activated carbons. *Carbon* **1999**, *37*, 1379–1389.
3. Figueiredo, J.L.; Pereira, M.F.R.; Freitas, M.M.A.; Órfão, J.J.M. Characterization of active sites on carbon catalysts. *Ind. Eng. Chem. Res.* **2007**, *46*, 4110–4115.
4. Bleda-Martínez, M.J.; Lozano-Castelló, D.; Morallón, E.; Cazorla-Amorós, D.; Linares-Solano, A. Chemical and electrochemical characterization of porous carbon materials. *Carbon* **2006**, *44*, 2642–2651.
5. Zhou, J.-H.; Sui, Z.-J.; Zhu, J.; Li, P.; Chen, D.; Dai, Y.-C.; Yuan, W.-K. Characterization of surface oxygen complexes on carbon nanofibers by TPD, XPS and FT-IR. *Carbon* **2007**, *45*, 785–796.
6. Marchon, B.; Tysoe, W.T.; Carrazza, J.; Heinemann, H.; Somorjai, G.A. Reactive and kinetic properties of carbon monoxide and carbon dioxide on a graphite surface. *J. Phys. Chem.* **1988**, *92*, 5744–5749.
7. Plaza, M.G.; Pevida, C.; Arias, B.; Fermoso, J.; Arenillas, A.; Rubiera, F.; Pis, J.J. Application of thermogravimetric analysis to the evaluation of aminated solid sorbents for CO_2 capture. *J. Therm. Anal. Calorim.* **2008**, *92*, 601–606.
8. Keramati, M.; Ghoreyshi, A.A. Improving CO_2 adsorption onto activated carbon through functionalization by chitosan and triethylenetetramine. *Phys. E Low Dimens. Syst. Nanostruct.* **2014**, *57*, 161–168.

9. Vuković, G.; Marinković, A.; Obradović, M.; Radmilović, V.; Čolić, M.; Aleksić, R.; Uskoković, P.S. Synthesis, characterization and cytotoxicity of surface amino-functionalized water-dispersible multi-walled carbon nanotubes. *Appl. Surf. Sci.* **2009**, *255*, 8067–8075.
10. Vuković, G.D.; Marinković, A.D.; Čolić, M.; Ristić, M.Đ.; Aleksić, R.; Perić-Grujić, A.A.; Uskoković, P.S. Removal of cadmium from aqueous solutions by oxidized and ethylenediamine-functionalized multi-walled carbon nanotubes. *Chem. Eng. J.* **2010**, *157*, 238–248.
11. Boehm, H. Surface oxides on carbon and their analysis: A critical assessment. *Carbon* **2002**, *40*, 145–149.
12. Moreno-Castilla, C.; Carrasco-Marín, F.; Mueden, A. The creation of acid carbon surfaces by treatment with $(NH_4)_2S_2O_8$. *Carbon* **1997**, *35*, 1619–1626.
13. Laginhas, C.; Nabais, J.M.V.; Titirici, M.M. Activated carbons with high nitrogen content by a combination of hydrothermal carbonization with activation. *Microporous Mesoporous Mater.* **2016**, *226*, 125–132.
14. Dubinin, M.M. Water vapor adsorption and the microporous structures of carbonaceous adsorbents. *Carbon* **1980**, *18*, 355–364.
15. Do, D.D.; Do, H.D. A model for water adsorption in activated carbon. *Carbon* **2000**, *38*, 767–773.
16. Dubinin, M.; Serpinsky, V. Isotherm equation for water vapor adsorption by microporous carbonaceous adsorbents. *Carbon* **1981**, *19*, 402–403.
17. Stoeckli, F. Recent developments in Dubinin's theory. *Carbon* **1998**, *36*, 363–368.
18. Horikawa, T.; Sekida, T.; Hayashi, J.; Katoh, M.; Do, D.D. A new adsorption–desorption model for water adsorption in porous carbons. *Carbon* **2011**, *49*, 416–424.
19. Do, D.D.; Junpirom, S.; Do, H.D. A new adsorption–desorption model for water adsorption in activated carbon. *Carbon* **2009**, *47*, 1466–1473.
